# What predicts the severity of alcohol use and related problems among young people presenting to emergency department or crisis support care?

**DOI:** 10.1186/1940-0640-10-S2-O33

**Published:** 2015-09-24

**Authors:** Catherine A Quinn, Leanne Hides

**Affiliations:** 1 Centre for Youth Substance Abuse Research, School of Psychology & Counselling, Institute of Health & Biomedical Innovation, Queensland University of Technology, Brisbane, Australia

## Background

Harmful and hazardous drinking is endemic among young people. In recent decades, there have been dramatic increases in the number of young people presenting to emergency departments with alcohol-related injuries and illnesses (inc. intoxication). Yet, little is known about what predicts the severity of alcohol use and related problems in these traditionally non-treatment seeking samples of young people.

This paper identifies what treatment orientated factors, including readiness to change, self-efficacy to reduce heavy drinking and coping strategies, relate to alcohol use and -related problems in young people presenting to an emergency department or crisis support care.

## Material and methods

Participants were 331 (52% female) young Australians aged 16-25 years (Mage = 20.3 years), who had come into contact with an emergency department (17.2%) or crisis support care (82.8%) with alcohol related injuries or illnesses and were referred to a brief alcohol intervention trial.

## Results

The average number of standard drinks consumed on the night of contact was 14.72 (SD = 9.04). A high proportion had not used illicit substances (78.2%) on the night of contact, had low psychological distress (61%), were precontemplative (90.8%), and had high confidence in their ability to stop themselves drinking heavily in most circumstances, except social situations. Regression analyses revealed that confidence to stop drinking was inversely related to alcohol problems and the number of drinks consumed on the night of contact. Readiness to change and emotion-oriented coping were positively related to alcohol-related problems.

## Conclusions

Despite engaging in risky alcohol use and experiencing alcohol-related problems, this sample of young people were quite confident in their ability to control their drinking. Potential targets for brief interventions will be discussed.

